# Retraction Note: *PPP1*, a plant-specific regulator of transcription controls Arabidopsis development and *PIN* expression

**DOI:** 10.1038/s41598-021-87406-5

**Published:** 2021-04-08

**Authors:** René Benjamins, Elke Barbez, Martina Ortbauer, Inez Terpstra, Doris Lucyshyn, Jeanette Moulinier-Anzola, Muhammad Asaf Khan, Johannes Leitner, Nenad Malenica, Haroon Butt, Barbara Korbei, Ben Scheres, Jürgen Kleine-Vehn, Christian Luschnig

**Affiliations:** 1 Department of Applied Genetics and Cell Biology, University of Natural Resources and Life Sciences, Vienna (BOKU), Muthgasse 18, 1190 Wien, Austria; 2 Plant Developmental Biology, Wageningen University Research, 6708 PB Wageningen, The Netherlands; 3 Swammerdam Institute for Life Sciences, Faculty of Science, University of Amsterdam, 1090 GE Amsterdam, The Netherlands; 4 Present Address: Syngenta Seeds B.V., Westeinde 62, 1601 BK Enkhuizen, The Netherlands; 5 Present Address: Gregor Mendel Institute of Molecular Plant Biology, Dr. Bohr-Gasse 3, 1030 Vienna, Austria; 6 Present Address: Department of Bioinformatics and Biotechnology (BNB), Government College University, Faisalabad (GCUF), Allama Iqbal Road, Faisalabad, 38000 Pakistan; 7 Present Address: Faculty of Science, Department of Molecular Biology, University of Zagreb, Horvatovac 102a, 10000 Zagreb, Croatia

Retraction of: *Scientific Reports* 10.1038/srep32196, published online 24 August 2016

The Authors have retracted this Article.

In follow-up experiments on this work, we noticed a mix up of transgenic lines expressing N- and C-terminally tagged *PPP1* reporter constructs under the 35S promoter. All the lines presented in the manuscript in Figure 4i–n correspond to the identical N-terminally tagged *35S::GFP:PPP1* expression cassette, and the GFP signal localizes to nucleus and cytoplasm. Expression of this construct results in a limited rescue of the *ppp1-476* seedling phenotypes. However, the level of complementation is not comparable to complementation by a C-terminally GFP-tagged version of the protein that is in fact localized to the chloroplast, as reported by Manavski et al. [1]. As such, we are unable to support the conclusions presented as a nuclear regulator of *PIN* expression.

René Benjamins, Elke Barbez, Martina Ortbauer, Inez Terpstra, Doris Lucyshyn, Jeanette Moulinier-Anzola, Muhammad Asaf Khan, Johannes Leitner, Nenad Malenica, Barbara Korbei, Ben Scheres, Jürgen Kleine-Vehn & Christian Luschnig agree with the retraction and its wording. Haroon Butt did not respond to the correspondence about the retraction.
